# Early postoperative mortality after simultaneous or staged bilateral primary total hip arthroplasty: an observational register study from the swedish Hip arthroplasty register

**DOI:** 10.1186/s12891-015-0535-0

**Published:** 2015-04-08

**Authors:** Anne Garland, Ola Rolfson, Göran Garellick, Johan Kärrholm, Nils P Hailer

**Affiliations:** Department of Orthopaedics, Visby Hospital, Visby, Sweden; Swedish Hip Arthroplasty Register, Gothenburg, Sweden; Department of Orthopaedics, Institute of Clinical Sciences, The Sahlgrenska Academy, University of Gothenburg, Gothenburg, Sweden; Department of Orthopaedics, Institute of Surgical Sciences, Uppsala University Hospital, Uppsala, Sweden; Harris Orthopaedic Laboratory, Massachusetts General Hospital, Harvard Medical School, Boston, USA

**Keywords:** Postoperative mortality, Perioperative mortality, Simultaneous bilateral total hip arthroplasty, Register, Registry, Total hip replacement, One-stage bilateral THR/THA, Two-stage bilateral THR/THA

## Abstract

**Background:**

Approximately a fifth of all total hip arthroplasty (THA) patients suffers from bilateral osteoarthritis of the hip. It is unclear whether mortality risks differ between simultaneous bilateral THA and staged bilateral THA. We investigated mortality after simultaneous THA compared with staged bilateral THA in the largest cohort hitherto reported.

**Methods:**

The 42,238 patients reported to have received bilateral primary THA from 1992 to 2012 in the Swedish Hip Arthroplasty Register were included. Tumours and fractures as underlying diagnoses were excluded. The time interval between the first and second THA was divided into four categories or treated as a continuous variable. Unadjusted survival was calculated according to Kaplan-Meier and adjusted Cox regression models were fitted in order to calculate crude and adjusted hazard ratios (HR) for the risk of death within different time frames.

**Results:**

Patients selected for simultaneous bilateral surgery were younger, more often male, and had lower ASA (American Society of Anesthesiologists) class than patients receiving staged procedures. The adjusted 90-day mortality after the second procedure did not differ between the four investigated groups (simultaneous bilateral [HR 1.3, CI 0.5-3.3], surgeries within 6 months [HR 1.1, CI 0.6-2.0], surgeries between 7 and 12 months [HR 0.7, CI 0.4-1.2], with second surgery after >12 months as the reference group). For patients older than 75 years, men, patients with ASA class 3 or above, and for patients with rheumatoid arthritis (RA) the 90-day mortality was increased. The unadjusted risk of implant revision of any hip was slightly higher for patients with simultaneous bilateral THA compared to those with staged procedure within one year, but after adjustment for age, gender, diagnosis and implant fixation these differences were no longer statistically significant.

**Conclusion:**

There were no clinically relevant differences in early postoperative mortality between simultaneous and staged bilateral surgery in healthy patients. Advanced age, RA, a high ASA class and male sex increased the risk of death within 90 days. There may be an issue with enhanced risk of implant revision in patients with simultaneous bilateral THA that needs to be explored further.

## Background

A substantial proportion of patients receiving a primary total hip arthroplasty (THA) suffers from bilateral osteoarthritis (OA) of the hip, and in Sweden approximately 17% of THA patients have symptoms motivating insertion of a contralateral THA at some point [1]. Bilateral disease may already be manifest when patients undergo their first THA, and in such cases either simultaneous or staged bilateral surgery can be performed.

THA is considered a safe procedure. The early postoperative mortality is low and has decreased over the last years, and a systematic review concluded that the average 90-day mortality after THA was 0.7% [2,3]. Simultaneous bilateral surgery is also described as feasible and safe. Excellent functional results after simultaneous bilateral surgery were already reported by Jaffe and Charnley in 1971 [4] and early postoperative mortality after this procedure is reported to be low. 90-day mortalities between 0.14 and 0.16% [5,6] and a peri-operative mortality (without specification of the exact time frame) of 0.31% vs. 0.18% when compared with unilateral surgery [7] are reported. Putative benefits of simultaneous bilateral THA include a single hospital stay, a shorter rehabilitation period, higher patient satisfaction, and lower costs per patient [8].

There remain however concerns about the safety of simultaneous bilateral THA procedures: A higher amount of haemorrhage, an increased number of thromboembolic events and cardiopulmonary complications, and reduced range of motion have been described after simultaneous bilateral operations [9-11].

A large number of patients is needed in order to address the issue of early postoperative mortality and morbidity. Assuming that 90-day mortality is 0.7% after THA [3], 1,346 patients with simultaneous bilateral THA and 13,460 controls would be required in order to detect an increase in mortality by a factor of 2 (alpha = 0.05, power = 80%; [12,13]). Given these numbers, many of the cited studies seem underpowered to detect clinically relevant differences in early postoperative mortality, since the reported study populations range from 35 to 6,258 [6,7,14-17] with the exception of one register study from New Zealand [18]. In addition, mortality after simultaneous bilateral THA has most commonly been investigated using unilateral surgery for comparison, and not staged bilateral surgery, which would be the relevant control group [6,7,16]. Thus, there is a lack of knowledge about the early postoperative mortality after simultaneous bilateral THA compared with staged bilateral procedures in larger cohorts.

The Swedish Hip Arthroplasty Register (SHAR) with nationwide individualised information on THA surgery combined with dates of death for deceased patients is a suitable platform to investigate the safety of simultaneous bilateral THA. We evaluated if there is an increased risk of early mortality after this procedure compared with staged bilateral THA.

## Methods

### Source of data

Data was obtained from the SHAR database. THA performed in Sweden have been reported to this register since 1979, initially as aggregated data based on hospital units and since 1992 as individualized data based on personal identification numbers. In Sweden all citizens have a personal identification number that is used in every contact with healthcare providers. Since the investigated endpoint was postoperative mortality the analyses take their starting point in 1992. The SHAR has been repeatedly validated and on national level the completeness of registrations has been stable around 96-98% [1,19,20] although some studies have reported lower completeness for the endpoint revision surgery [1,21]. Registration of height and weight to calculate Body Mass Index (BMI) and American Society of Anesthesiologists (ASA) class were included in the SHAR from 2008. The completeness with respect to these parameters has improved over the years and in 2012 it reached 94.7% for BMI and 97.5% for ASA class [1].

Patient-reported outcomes data were not included in the analysis. Neither socioeconomic factors nor comorbidities are registered in the SHAR and were therefore not investigated.

The Regional Ethical Review Board in Gothenburg (2013: 360–13) approved our study. All participants have received written information about the SHAR and given the choice not to participate in the registry or associated research. Written informed consent for participation has not been obtained. This is in consistency with the Swedish Patient Data Law from 2009.

### Study population

During the study period (1992–2012) 42,238 THA had been performed as bilateral THA and thus 42,238 patients (22.1% of all patients registered during the study period) with bilateral THA were included in our analyses. In this study population 25,115 (59.5%) were women. The most common underlying diagnosis was OA (n = 38,779, 92%), the most common age group was 60–75 years (n = 23,436, 55.5%), and the most common fixation method during the second procedure was cemented (n = 33,836, 80.4%; Table 1). The age grouping is the same as that is used in the annual reports from the SHAR. Since information on BMI and ASA was not included in the SHAR until 2008 a subcohort of 15,226 patients included from 2008 and onwards was evaluated in a separate analysis (Table 2). Patients with femoral neck fractures and insertion of THA due to primary or metastatic malignancy were excluded from the study population. Revision arthroplasties were also not included in the analyses.

**Table 1:** **Demographics for the 42**,**238 individuals with bilateral THA operated 1992**-**2012**

	**Op same day (n = 1,680)**		**Op ≤ 6 months (n = 4, 867)**		**Op 7–12 months (n = 7,809)**		**Op > 12 months (n = 27, 882)**		**p-value**
	**n**	**%**	**n**	**%**	**n**	**%**	**n**	**%**	
**Age**									
<50	308	18.3	357	7.3	454	5.8	831	3.0	<0.001^a^
50-59	424	25.2	823	19.0	1,278	16.4	3,052	10.9	
60-75	788	46.9	2,792	57.4	4,499	57.6	15,357	55.1	
>75	160	9.5	795	16.3	1,578	20.2	8,642	31.0	
**Diagnoses**									
OA	1,346	80.4	4,309	88.8	7,112	91.4	26,012	93.4	<0.001^a^
RA	193	11.5	279	5.7	316	4.1	1,033	3.7	
Other^b^	136	8.1	266	5.5	351	4.5	819	2.9	
**Sex**									
Male	767	45.7	2,074	42.6	3,176	40.7	11,106	39.8	<0.001^a^
Female	913	54.3	2,793	57.4	4,633	59.3	16,776	60.2	
**Fixation**									
Cemented	931	55.8	3.697	76.5	6,224	80.1	22,984	82.6	<0.001^a^
Uncemented	372	22.3	563	11.6	686	8.8	2,147	7.7	
Other^c^	364	21.8	575	11.9	856	11.0	2,683	9.6	

^a^Chi - square.

^b^Sequelae from childhood hip disease, femoral head necrosis, secondary OA.

^c^Hybrid, reversed hybrid, and resurfacing.

All data in this table concerns the second operation.

**Table 2:** **The subcohort of 15,226 individuals with information on ASA class and BMI**

	**Op same day (n = 450)**	**Op < 6 months (n = 1,598)**	**Op 6–12 months (n = 2,286)**	**Op > 12 months (n = 10,892)**	**p-value**
**BMI X (SD)**	26.9(4.7)	27.3(5.7)	27.5(4.8)	27.5(5.0)	0.050^a^
**ASA n (%)**					
**1**	157(33.5%)	472(29.1%)	624(26.5%)	2,304(20.8%)	<0.001^b^
**2**	250(53.3%)	974(60.1%)	1,398(59.4%)	6,823(61.5%)	
**3-5**	62(13.2%)	174(10.7%)	331(14.1%)	1,967(17.7%)	

^a^ANOVA.

^b^Chi-square.

15,226 individuals with ASA and BMI registered. 15,536 had ASA, and 15,226 had BMI registered.

For comparative reasons patients who had had a unilateral THA during the same time period (1992–2012) were also analysed. This cohort consisted of 148,718 patients (87.9% of all patients registered during the study period). In the unilaterally operated population 85,102 (57.2%) were women. The most common underlying diagnosis was OA (n = 131,405; 89.3%), the most common age group was 60–75 years (n = 76,473; 51.4%), and the most common fixation method during the THA procedure was cemented (n = 121,393; 81.6%).

### Terminology

The term “simultaneous THA” is commonly used for one-stage surgery although the hips are commonly operated upon sequentially but during one anaesthesia. This definition of simultaneous is used in our study. The most common diagnoses in our study population were primary OA and inflammatory arthritis (RA; rheumatoid arthritis and related inflammatory joint diseases) (Table 1). Other diagnoses such as childhood hip disease, femoral head necrosis and secondary arthritis were more rare and were therefore grouped together as “other”.

Most commonly both the cup and stem were cemented followed by fixation without cement. Hybrid fixation modes including one cemented and one uncemented component (hybrids, reversed hybrids, resurfacings) were less frequently used and therefore classified into one group (“other”, Table 1). ASA classes 1 and 2 were most common, ASA class 3 was less common, and class 4 and 5 was highly infrequent, why the latter 3 categories were grouped together as ASA score 3–5. Age, fixation method, BMI, and ASA class were measured at the time of the second operation.

The time interval between the first and second THA was either treated as a continuous variable or divided into four categories (simultaneous bilateral surgery, ≤ 6 months [1–179 days], 7–12 months [180–365 days], and >12 months [>365 days] between surgeries).

We investigated 30-day-, 90-day-, 10-year- and overall mortality after the second surgery (index surgery). Our primary endpoint was 90-day mortality since this parameter captures both perioperative deaths, early postoperative and delayed postoperative deaths such as those caused by thromboembolic events [5,6]. 30-day-, 10-year- and overall mortality after the second surgery were secondary endpoints. Revision surgery of any hip after the second THA surgery was also explored as an additional endpoint.

### Statistics

We adhered to the guidelines on statistical analyses of register data [22,23]. Follow-up started on the day of the second surgery and ended on the day of death, emigration, or December 31st 2012, whichever came first. Continuous data were described using means, medians, and ranges, and 95% confidence intervals (CI) were used to describe estimation uncertainty. Categorical data were summarized in cross-tables and the Chi-square test or Fisher’s exact test were used to investigate differences between groups. Kaplan-Meier survival analysis was performed to calculate unadjusted survival for different time periods (see above). Cox regression models were fitted for each covariate at a time in order to calculate crude hazard ratios (HR) with CI, and covariates were subsequently included in multiple regression models in order to calculate adjusted HR with CI. The choice of covariates included in multiple regression models was based upon assessment of relevance and non-interference using directed acyclic graphs as previously suggested [24]. For exploratory analyses the material was stratified into different periods of time (1992–1996, 1997–2001, 2002–2006, 2007–2011 and 2012). The resulting five subcohorts were analysed separately as above. Separate models were also fitted for a subcohort of patients where information on BMI and ASA was available. Model assumptions were investigated by calculating and plotting the correlation coefficient between transformed survival time and the scaled Schoenfeld residuals. The level of significance was set at p < 0.05. The R software (package 3.0.2 [25]) and SPSS (version 22) were used for analyses.

## Results

63 patients (0.1%) died within 30 days after second surgery, 125 (0.3%) within 90 days and 6,178 (14.7%) within 10 years. Crude survival for the investigated groups is given in Table 3. The group of patients that underwent simultaneous bilateral surgery differed from the other groups with respect to prevailing diagnoses, type of fixation, and age. OA was the dominant underlying diagnosis (80.4%) but RA was more common among patients operated with simultaneous bilateral surgery than among those that underwent staged procedures. Patients selected for simultaneous bilateral surgery were younger and slightly more often males. Alternatives to cemented fixation were chosen more often in this group (See Table 1).

**Table 3:** **The four investigated groups and time intervals, with crude survival percentages and 95% confidence intervals for each group**

	**30 days**		**90 days**		**10 years**	
	**Survival**	**CI (95%)**	**Survival**	**CI (95%)**	**Survival**	**CI (95%)**
**Time interval**						
Simultaneous	99.8	99.6-100.0	99.7	99.4-100.0	82.7	80.4-85.1
≤6 months	99.8	99.7-99.9	99.7	99.6-99.9	78.1	76.5-79.|7
7-12 months	99.9	99.8-100.0	99.8	99.7-99.9	76.9	7|5.7-78.2
>12 months	99.8	99.8-99.9	99.7	99.6-99.7	71.5	70.7-72.3

### Early postoperative mortality

**Figure 1:**
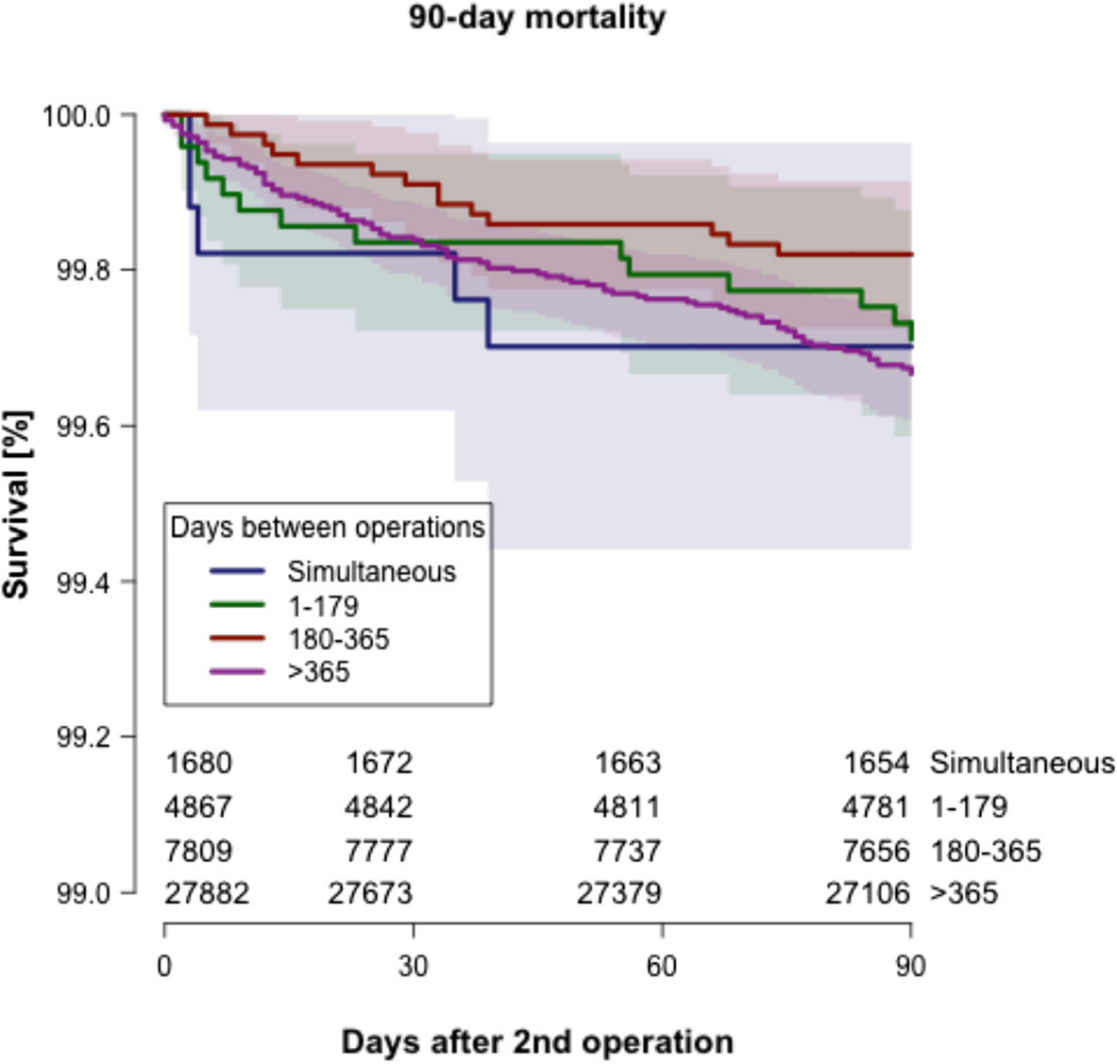
**Early mortality up to 90--days after second THA in bilaterally operated patients**. Unadjusted survival according to Kaplan---Meier. Shaded areas indicate 95% confidence intervals.

Unadjusted survival at different time points is given for the four investigated groups in Table 3 and unadjusted 90-day survival is depicted in Figure 1. For comparison, the unadjusted survival in the unilaterally operated control cohort was 99.4% (CI 99.3-99.4) 90 days after the index THA surgery.

For bilaterally operated patients, the risk of dying within 90 days after the second procedure was not different between the four investigated groups after adjustment for sex, age, diagnosis, and type of prosthesis fixation (Table 4). When time between surgeries was treated as a continuous variable the timing of surgery still had no influence on the adjusted risk of dying within 90 days (HR 1.0, CI 1.0-1.0). However, for patients over 75 years old and for men, the risk of dying within 90 days was increased (Table 4) and there was a doubled risk of dying within 90 days after index surgery for patients with RA (HR 2.3, CI 1.1-4.7). Analyses of the subcohort of patients among whom information on ASA class and BMI was available indicated that ASA class 3 and above was a risk factor for increased 90-day mortality after index surgery when compared with ASA class 1 (HR 8.2, CI 1.8-37.3, after adjustment for sex, type of prosthesis fixation, and BMI, see Table 5). Adding underlying diagnoses to the adjusted model (adjustment for sex, type of prosthesis fixation, and BMI) did not alter the 90-day mortality estimates for the subcohort with information on ASA class and BMI (data not shown).

**Table 4:** **Hazard ratio for 90-day mortality**

	**Crude**			**Adjusted**		
	**HR**	**CI (95%)**	**p**	**HR**	**CI (95%)**	**p**
**Time interval**						
Simultaneous	0.69	0.4-2.2	0.82	1.3	0.5-3.3	0.59
≤6 months	0.9	0.5-1.5	0.63	1.1	0.6-2.0	0.72
7-12 months	0.5	0.3-1.0	0.03	0.7	0.4-1.2	0.14
>12 months (ref)^a^	1.0	-	-	1.0	-	-
**Sex**						
Male	1.0	-	-	1.0	-	-
Female	0.7	0.5-1.0	0.06	0.6	0.4-0.9	0.01
**Age**						
<50	1.1	0.4-3.2	0.80	1.0	0.3-3.0	0.98
50-59	0.7	0.3-1.5	0.36	0.7	0.3-1.5	0.34
60-75 (ref)	1.0	-	-	1.0	-	-
>75	3.6	2.6-5.8	<0.001	3.8	2.6-5.6	<0.001
**Fixation**						
Cemented (ref)	1.0	-	-	1.0	-	-
Uncemented	0.5	0.2-1.1	0.09	0.9	0.4-2.2	0.80
Other^b^	0.7	0.4-1.3	0.27	1.1	0.6-2.2	0.75
**Diagnoses**						
OA (ref)	1.0	-	-	1.0	-	-
RA	1.7	0.9-3.3	0.13	2.3	1.1-4.7	0.02
Other^c^	0.4	0.1-1.7	0.24	0.6	0.1-2.4	0.44

Adjustment for sex, age, diagnosis, and type of prosthesis fixation.

^a^Ref: reference.

^b^Hybrid, reversed hybrid, and resurfacing.

^c^Sequelae from childhood hip disease, idiopathic femoral head necrosis, secondary OA.

Number of events 125.

**Table 5:** **Hazard ratios for 90-day mortality for the ASA class and BMI subcohort consisting of 15,226 individuals**

	**Crude**			**Adjusted**		
	**HR**	**CI (95%)**	**p**	**HR**	**CI (95%)**	**p**
**Time interval**						
Simultaneous	1.0	0.1-7.1	0.97	1.4	0.2-10.6	0.75
≤6 months	0.5	0.1-2.3	0.41	0.7	0.2-2.9	0.61
7-12 months	0.8	0.3-2.2	0.61	0.9	0.3-2.5	0.78
>12 months (ref)^a^	1.0	-	-	1.0	-	-
**BMI**	1.0	0.9-1.1	0.83	1.0	0.9-1.1	0.76
**ASA**						
1(ref)	1.0	-	-	1.0	-	-
2	2.3	0.7-7.7	0.19	3.0	0.7-13.0	0.15
3-5	6.6	1.9-22.9	0.003	8.2	1.8-37.3	0.006
**Sex**						
Male	1.0	-	-	1.0	-	-
Female	0.8	0.4-1.5	0.45	0.4	0.4-1.5	0.40
**Fixation**						
Cemented (ref)	1.0	-	-	1.0	-	-
Uncemented	0.4	0.1-1.6	0.18	0.5	0.1-2.0	0.30
Other^b^	0.7	0.3-2.1	0.56	0.8	0.3-2.4	0.73

Adjustment for sex, ASA, BMI, and type of prosthesis fixation.

^a^Ref: reference group.

^b^Hybrid, reversed hybrid, and resurfacing.

Number of events 32.

After adjustment for sex, age, diagnosis, and type of prosthesis fixation the HR for the risk of dying within the first 30 postoperative days after the second procedure was 2.0 (CI 0.6-6.6) for simultaneous surgery, 1.4 (CI 0.7-3.0) for surgery within 6 months, and 0.7 (CI 0.3-21.5) for surgery between 7 and 12 months with second surgery after more than 12 months as the reference group (Table 6).

**Table 6:** **Hazard ratio for 30-day mortality**

	**Crude**			**Adjusted**		
	**HR**	**CI (95%)**	**p**	**HR**	**CI (95%)**	**p**
**Time interval**						
Simultaneous	1.1	0.3-3.6	0.86	2.0	0.6-6.6	0.26
≤6 months	1.0	0.5-2.2	0.96	1.4	0.7-3.0	0.39
7-12 months	0.6	0.3-1.2	0.15	0.7	0.3-1.5	0.37
>12 months (ref)^a^	1.0	-	-	1.0	-	-
**Sex**						
Male	1.0	-	-	1.0	-	-
Female	0.8	0.5-1.3	0.38	0.7	0.4-1.2	0.16
**Age**						
<50	0.5	0.1-4.1	0.55	0.6	0.1-5.3	0.68
50-59	0.4	0.1-1.6	0.18	0.4	0.1-1.7	0.22
60-75 (ref)	1.0	-	-	1.0	-	-
>75	3.6	2.1-6.1	<0.001	3.8	2.2-6.5	<0.001
**Fixation**						
Cemented (ref)	1.0	-	-	1.0	-	-
Uncemented	0.5	0.1-1.5	0.21	1.0	0.3-3.6	0.97
Other^b^	0.4	0.1-1.3	0.12	0.7	0.2-2.4	0.59

Adjustment for sex, age, and type of prosthesis fixation. Stratified for diagnosis since proportional hazard was missing for this variable.

^a^Ref: reference.

^b^Hybrid, reversed hybrid and resurfacing.

Number of events 63.

### Overall mortality

The crude and adjusted overall risk of death for the four investigated groups (simultaneous, ≤ 6 months, 7–12 months and > 12 months) differed significantly between groups, with 10-year and overall mortality being lower in the groups of patients selected for simultaneous bilateral surgeries or surgeries within 1 year. The adjusted HR for the overall risk of death was 0.9 (CI 0.8-1.0) for simultaneous surgery, 0.9 (CI 0.8-1.0) for surgery within 6 months, and 0.9 (CI:0.9-1.0) for surgery between 7 and 12 months after adjustment for sex, age, diagnosis, and type of prosthesis fixation, and with second surgery more than 12 months after the first THA as the reference group (Table 7).

**Table 7:** **Hazard ratio for overall mortality**

	**Crude**			**Adjusted**		
	**HR**	**CI (95%)**	**p**	**HR**	**CI (95%)**	**p**
**Time interval**						
Simultaneous	0.6	0.5-0.6	<0.001	0.9	0.8-1.0	0.089
≤6 months	0.8	0.7-0.8	<0.001	0.9	0.9-1.0	0.0|32
7-12 months	0.8	0.8-0.8	<0.001	0.9	0.9-1.0	0.007
>12 months (ref)^a^	1.0	-	-	1.0	-	-
**Sex**						
Male	1.0	-	-	1.0	-	-
Female	0.8	0.8-0.8	<0.001	0.2	0.1-0.2	<0.001
**Age**						
<50	0.2|	0.2-0.3	<0.001	0.2	0.1-0.2	<0.001
50-59	0.3	0.3-0.4	<0.001	0.4	0.3-0.4	<0.001
60-75 (ref)	1.0	-	-	-	-	-
>75	3.1	3.0-3.3	<0.001	3.2	3.1-3.4	<0.001
**Fixation**						
Cemented (ref)	1.0	-	-	-	-	-
Uncemented	0.3	0.2-0.3	<0.001	0.7	0.6-0.8	<0.001
Other^b^	0.3	0.3-0.4	<0.001	0.7	0.6-0.7	<0.001
**Diagnoses**						
OA (ref)	1.0	-	-	1.0	-	-
RA	1.2	1.1-1.3	<0.001	2.2	2.0-2.4	<0.001
Other^c^	0.7	0.6-0.8	<0.001	1.5	1.3-1.7	<0.001

Adjustment for sex, age, diagnosis, and type of prosthesis fixation.

^a^Ref: reference.

^b^Hybrid, reversed hybrid and resurfacing.

^c^Sequelae from childhood hip disease, idiopathic femoral head necrosis, secondary OA.

Number of events 8267.

The overall 1-year mortality after the second THA was 1.2% (1,200 per 100,000) for all patients grouped together.

### Revision surgery

Revision surgery of either hip after the second THA surgery was slightly more common in simultaneously operated patients compared to the patients operated with a staged procedure [4-7,14-16] 240 [7.1%], surgeries within 6 months n 495 [5.1%], surgeries between 7 and 12 months n 828 [5.3%] and second surgery after >12 months n 3,573 [6.4%].

The unadjusted risk of revision for the simultaneously operated patients compared to the patients operated with staged procedure was [4-7,14-16]. When adjusting for sex, age, diagnosis and type of prosthesis fixation this difference in the risk estimates disappeared [4-7,14-16].

### Observation period as a covariate

We stratified the material into five subcohorts with respect to the year of surgery. The mortality was somewhat lower for the more recently operated patients compared to patients operated during earlier time periods (90-day unadjusted overall survival 1992–1996 was 99.7% [CI 99.5-99.9], 1997–2001 99.5% [CI99.4-99.7], 2002–2006 99.7% [CI 99.6-99.8], 2007–2011 99.8% [CI 99.7-99.9] and 2012 99.8% [CI 99.6-99.9]). Apart from that no relevant differences in mortality could be detected in the stratified material compared to the results obtained on the entire study population (data not shown).

Simultaneous surgery was more common early in the study period. 227 patients (6.4%) were operated simultaneously 1992–1996, 373 patients (4.9%) in 1997–2001, 500 patients (4.2%) in 2002–2006, 494 patients (3.2%) 2007–2011 and 86 patients (2.5%) were operated simultaneously in 2012. The time period was introduced as a covariate in a Cox regression model for 90-day mortality. After adjustment for sex, age, diagnosis and type of prosthesis fixation the risk of 90-day mortality was not statistically significantly different during the investigated time periods (1997–2001 [HR 1.3, CI 0.7-2.4], 2002–2006 [HR 0.8, CI 0.4-1.5], 2007–2011 [HR 0.5, CI 0.3-1.1], 2012 [HR 0.6, CI 0.2-1.5] with the first period 1992–1996 as reference).

### Exploratory analyses

Exploratory analyses showed that the type of hospital where the procedures were performed and the type of surgical approach did not influence the risk of death within 30 or 90 days after the second procedure (data not shown). Hybrids, reverse hybrids, and resurfacing arthroplasties were also analysed as separate modes of fixation instead of being grouped together, but this did not influence parameter estimates in a statistically significant manner (data not shown).

Instead of categorizing the time between surgeries into four groups we also investigated the time interval between the two THA procedures in each patient as a continuous variable. This analysis indicated that elapsed time between surgeries was not a statistically significant risk factor for death within 90 days after the second THA.

## Discussion

17% of patients receiving a primary THA in Sweden suffer from or will develop bilateral disease with symptoms motivating insertion of a contralateral THA [1]. Some are operated bilaterally on one day, which is considered a safe procedure. However, doubts regarding postoperative mortality remain since previous studies only included small populations or lacked a relevant control group [4-7,14-16].

In this observational study on the largest cohort hitherto reported we compare simultaneous bilateral THA with staged bilateral surgery, which is a more adequate reference group when compared to patients that only underwent one THA. The results indicate that early mortality after simultaneous bilateral surgery is not increased when compared with staged procedures. We also find that patients selected for simultaneous procedures are younger and healthier than those that receive staged surgery.

### Postoperative mortality after THA

THA is a relatively safe procedure and early postoperative mortality is low, varying between 0.1% to 0.7% (30 day mortality 0.1% [26], 90 day mortality 0.29% [2] and 0,7% [1]). In Sweden, the 90-day mortality after THA, with unilateral and bilateral together, has been reported to be 0.7%. The reason for this slightly increased mortality in the Swedish population in comparison to some other studies could be explained by the inclusion of patients with femoral neck fractures in the Swedish records. Differing patient demography or varying completeness of mortality records can also contribute to this phenomenon [1]. However, in a systematic review on the subject of mortality following THA Singh et al. [3] describe a 90-day mortality of 0.7% which supports the figures reported from the SHAR.

The 1-year mortality after the second THA was 1,2% (1,200 per 100,000) for all patients together. This figure can be compared with the overall Standardized Death Rate (SDR) of the Swedish population that was 514 per 100,000 in 2010 [27], indicating a higher 1-year mortality in THA patients when compared with the average population. This finding is in agreement with the fact that THA patients are older than the average population.

RA as the underlying diagnosis for THA surgery, male gender, and an age above 75 years at the time of second surgery were associated with an increased risk of death within 90 days. These findings were expected. Due to their comorbidities patients with RA represent a vulnerable group, and earlier work indicates a higher complication rate for RA patients although they are generally younger at time of surgery and more often female [28-30].

The larger proportion of men among patients selected for simultaneous bilateral surgery in our material is notable since our analyses and other studies indicate that male sex is associated with an increased risk of early postoperative death when compared with females [26,31,32]. The reason why men are more often selected for bilateral surgery is unclear. From a general perspective there are no reasons to believe that men are in a greater need of pain-free hips. In the SHAR males are operated at a younger age than females, which may suggest an earlier debut of symptoms or a gender influence on indications. Nonetheless, it might be that simultaneous bilateral THA surgery is underutilized in females.

### Strengths and weaknesses of this study

Many of the cited studies seem underpowered to detect clinically relevant differences in early postoperative mortality given the numbers mentioned above [5-7]. In addition, mortality after simultaneous bilateral THA is commonly compared with mortality after unilateral surgery [6,7,16] but only very rarely with mortality after staged bilateral surgery (Saito S, Tokuhashi Y, Ishii T, Mori S, Hosaka K and Taniguchi S [15]; study cohort of n = 178 THA, Shih CH and Ho WB [14]; n = 70 THA, Hooper GJ, Hooper NM, Rothwell AG and Hobbs T [18]; study cohort n = 13,151 THA), which would be the relevant control group. A strength of our study is the relatively large size of the investigated cohort, giving the opportunity to detect clinically relevant differences in early mortality after THA.

In this study on the largest cohort hitherto reported we compare simultaneous bilateral THA with staged bilateral surgery. The time interval between the first and second procedure in staged THA was divided into four categories (simultaneous bilateral surgery, second surgery within 6 months, between 7 to 12 months, and second surgery after more than 12 months). Patients who undergo 2 THA within 12 months can be considered likely to have had bilateral OA when the decision regarding the first surgery was made and might have been offered a simultaneous procedure [33]. Patients who undergo 2 THA with more than 12 months in between can be considered less likely to have been eligible for a simultaneous procedure when the decision regarding the first surgery was made. These arbitrary categories can be questioned, although they were chosen in line with previous work on this topic [33].

As in other observational studies the issue of selection bias has to be considered. Patients selected for simultaneous bilateral surgery were younger, were more often men, had lower ASA class, more often had other underlying diagnoses than OA, and were more often selected for uncemented or other fixation methods. Thus, the investigated groups were not equal with respect to these aspects. From a clinical point of view the most relevant approach would be to compare patients with bilateral symptoms and with the same type of hip disease who at their first visit are judged to be subjected to either a simultaneous bilateral operation or a staged procedure. A comparison of those operated simultaneously with those operated within 6 or perhaps even 12 months would be interesting. Patients planned for staged surgery may suffer from complications postponing the planned second stage or preventing the patient from having the second operation. Some may even die before their second hip is operated. In our study we calculated mortality after the second procedure, consequently the postoperative mortality was slightly underestimated because those who died between the first and second operation were excluded. Theoretically one could estimate this excess mortality rate, but we have refrained from doing so due to the risk of including too many uncertainties. This is a drawback of our study but nonetheless speaks in favour of simultaneous bilateral procedure since the mortality rate in the staged group is a more or less conservative approximation.

The SHAR does not include information on medical comorbidities, causes of death, and causes of readmission after THA surgery. Medical comorbidities influence the clinical outcome after THA surgery [34] and could affect peri- and postoperative mortality [2,30,35,36]. Ideally our analyses should have been adjusted for medical comorbidity, and further studies based on combined data from the SHAR and the national inpatient register would therefore be of great value.

The question of selection bias has to be considered also when analysing the revision rates of this study. We found slightly increased revision rates in simultaneously operated patients compared to patients who had had a staged procedure. An observandum is that patients selected for a simultaneous procedure more often were selected for uncemented or other fixation methods compared to patients selected for a staged procedure.

## Conclusion

Our results based on register data on 42,238 patients show no relevant difference in perioperative mortality between simultaneous and staged bilateral surgery in healthy patients with symptoms motivating this type of procedure. Advanced age, RA, high ASA class and male sex increased the risk of death, which should be taken into consideration when making the choice between simultaneous or staged bilateral THA.
