# Erratum to: Early postoperative mortality after simultaneous or staged bilateral primary total hip arthroplasty: an observational register study from the Swedish Hip Arthroplasty Register

**DOI:** 10.1186/s12891-015-0717-9

**Published:** 2015-09-28

**Authors:** Anne Garland, Ola Rolfson, Göran Garellick, Johan Kärrholm, Nils P. Hailer

**Affiliations:** Department of Orthopaedics, Visby Hospital, Visby, Sweden; Swedish Hip Arthroplasty Register, Gothenburg, Sweden; Department of Orthopaedics, Institute of Clinical Sciences, The Sahlgrenska Academy, University of Gothenburg, Gothenburg, Sweden; Department of Orthopaedics, Institute of Surgical Sciences, Uppsala University Hospital, Uppsala, Sweden; Harris Orthopaedic Laboratory, Massachusetts General Hospital, Harvard Medical School, Boston, USA

**Erratum:**

The original version of this article unfortunately contained a mistake in the section entitled “Revision surgery”. The corrected text is given below:

**Corrected text:**

(Page 5: Results - Revision surgery)

Revision surgery of either hip after the second THA surgery was slightly more common in simultaneously operated patients compared to the patients operated with a staged procedure (Simultaneous surgeries 240 [7.1 %], surgeries within6 months n 495 [5.1 %], surgeries between 7 and 12 months n 828 [5.3 %] and second surgery after >12 months n 3,573 [6.4 %]).

The unadjusted risk of revision for the simultaneously operated patients compared to the patients operated with staged procedure was [HR 1.6, CI 1.3-1.9] (surgeries within 6 months [HR 1.2, CI 1.1-1.4], surgeries between 7 and 12 months [HR 1.1, CI 1.0-1.3] with second surgery after >12 months as the reference group). When adjusting for sex, age, diagnosis and type of prosthesis fixation this difference in the risk estimates disappeared (simultaneous bilateral [HR 1.1, CI 0.9-1.4], surgeries within 6 months [HR 1.1, CI 1.0-1.3], surgeries between 7 and 12 months [HR 1.1, CI 1.0-1.2] with second surgery after >12 months as the reference group).
